# Object Identification for Task-Oriented Communication with Industrial Robots

**DOI:** 10.3390/s20061773

**Published:** 2020-03-23

**Authors:** Adam Rogowski, Piotr Skrobek

**Affiliations:** Faculty of Production Engineering, Warsaw University of Technology, ul. Narbutta 86, 02-524 Warszawa, Poland

**Keywords:** image recognition, industrial robots, man-machine communication

## Abstract

To make the human-robot collaboration effective, it may be necessary to provide robots with “senses” like vision and hearing. Task-oriented man-machine speech communication often relies on the use of abstract terms to describe objects. Therefore it is necessary to correctly map those terms into images of proper objects in a camera’s field of view. This paper presents the results of our research in this field. A novel method for contour identification, based on flexible editable contour templates (FECT), has been developed. We demonstrate that existing methods are not appropriate for this purpose because it is difficult to formulate general rules that humans employ to rank shapes into proper classes. Therefore, the rules for shape classification should be individually formulated by the users for each application. Our aim was to create appropriate tool facilitating formulation of those rules as it could potentially be a very labor-intensive task. The core of our solution is FCD (flexible contour description) format for description of flexible templates. Users will be able to create and edit flexible contour templates, and thus, adjust image recognition systems to their needs, in order to provide task-oriented communication between humans and robots.

## 1. Introduction

Interaction between man and machine plays an increasingly important role in modern industry. A good example is collaborative robotics. Collaborative robots participate in various tasks where manipulation of small parts or intricate motions are involved. This includes machine tending [1], assembly [2], painting [3], coating [4], and many others. Direct cooperation between humans and industrial robots, in order to pursue common goals enables synergistic effects to be achieved through the combination of the robot’s precision and strength with human dexterity [5]. Many researchers deal with various aspects of collaborative robotics, e.g., human-oriented robot design [6], robot programming [7,8], sensory data sharing in multirobot systems [9], human trust in safety measures [10], and many others. An important topic of research in this area is human-machine communication. Efficient interaction plays a crucial role in the Industry 4.0 concept because it helps the workers realize their full potential, and adopt the role of strategic decision-makers and flexible problem-solvers [11]. As shown in a survey on human-robot collaboration in industrial settings [12], the most popular advanced means of human-robot communication are speech and gestures.

This kind of interaction requires robots to be equipped with artificial senses, such as hearing and vision. As far as controlling industrial robots by speech is concerned, several laboratory experiments were conducted at various universities [13,14,15,16]. An important advantage of speech is that it does not involve operator’s hands. For example, Zinchenko et al. developed a voice control system for a surgical robot [17]. 

As far as vision is concerned, its application in industrial robotics makes it possible to achieve various goals like real-time collision avoidance [18], evaluation of imperfections in manufactured parts [19], determination of target objects for robot picking tasks [20], and many others. There are also numerous reports of research regarding gesture recognition in the context of robot programming and control. For example, Tsarouchi et al. deal with industrial robot programming, based on detection of human motions using visual sensors [21]. A very popular topic of research is the combination of both methods (speech and gestures) [22,23,24].

However, the parallel use of speech and image recognition does not have to be restricted to speech-gesture combination. As stated by Pires [25], an important challenge for industrial robotic cell programming is to develop methodologies that could help users to control and program a robot, with a higher level of abstraction from robot language. High levels of abstraction may also relate to the description of objects manipulated by the robot. Visual information, shared by a speaker (human) and listener (robot), allow the speaker to produce inexplicit utterances [26]. In order to provide effective speech communication with industrial robots, the voice commands should be formulated in a task-oriented and concise manner, e.g., “Reach me the wrench”. It means that objects manipulated by robots are described using abstract terms (like “wrench”) that cannot be directly assigned numerical values to generate robot positioning instructions. Unfortunately, data describing abstract objects and data describing robots’ environment are rarely associated with each other [27].

So far, little attention has been paid to vision-based object recognition aimed at mapping the abstract terms into objects in camera’s field of view. An example of such research is the work by Tasevski et al. [28] where automatic speech recognition was used in order to control an industrial robot grasping the objects specified by oral expressions. However, their solution refers only to the recognition of simple geometric figures of various colors. Our aim is to fill the gap in research and develop a general solution in the specific domain of industrial robotics. This involves several aspects, including voice command analysis [15], shape recognition, as well as formulation of identification rules for mapping an abstract term into an object described by several contours derived from image recognition system. The current paper presents an essential part of our research that deals with the identification of individual contours for task-oriented human-robot communication in industrial scenario. We describe a novel method for contour identification that is based on flexible editable contour templates (FECT). One essential quality is that it allows the classification of contours of different dimensions and/or shapes to one group (corresponding to certain abstract term). Therefore, it may be employed in task-oriented communication between humans and industrial robots, and in particular, in voice communication. As such interactions are characteristic mainly for collaborative robotics, this domain is expected to be the main application area for our method.

Generally, there are two main aspects of vision application in industrial collaborative robotics. The first one refers to monitoring the regions surrounding the robot (usually with respect to possible human-robot collision), as well as to the recognition of operator’s gestures. These issues were the subject of extensive research [29,30]. Our method is related to the second aspect that has been paid little attention to: Recognition of objects (tools, workpieces) based on their description by abstract terms.

The remainder of this article is organized as follows. Section 2 contains the review of shape recognition and classification methods. On this background, it explains the main contribution of the paper in detail. General conception of FECT for contour classification is presented in Section 3. The core of this conception is FCD (flexible contour description) format developed for the purpose of creating the templates. Shape classification algorithm is presented in Section 4. Experimental results are discussed in Section 5. Finally, Section 6 contains conclusions and plans for the future.

## 2. Shape Recognition and Classification Methods

As stated by Hernández et al., the techniques and methods that detect objects depend on the application environment [31]. There are systems developed to work on mobile robots, performing navigation tasks and environment categorization, where accuracy is not the most important factor. The examples are systems for semantic navigation of mobile robots [32,33]. Better recognition accuracy must be provided, e.g., by multisensory system for service robots used in automatic harvesting of fruits [34]. However, the highest requirements must be fulfilled by industrially-oriented systems. In order to properly classify the images of objects like workpieces and tools, even little details cannot be neglected. Admittedly, some industrially-oriented systems make use of basic geometric properties, such as area and moments of inertia only [35] or additionally contour perimeter and dimension of minimal enclosing circle [28]. However, such simple methods are sufficient only when a very narrow spectrum of strictly determined objects is expected. Generally, a detailed description of shape is needed to compare the objects with their templates.

There is a big number of shape representation techniques used in various image processing methods, generally divided into contour-based and region-based ones [36]. Contour-based techniques, include chain codes, polygon approximation, B-spline approximation, shape signature, Fourier descriptors, wavelet descriptors, and others. As far as region-based techniques are concerned, the geometric moments, moment invariants, Zernike moments, Legendre moments, convex hulls, and many others may be applied.

The chain codes describe contours by a sequence of unit-size line segments with given orientation. They can be used for image compression [37], recognition of monotonic parts in contours [38], and even for face recognition [39]. Contour discretization obtained by polygon approximation lets lower the computational cost in subsequent analysis [40]. An interesting research, dealing with polygon recognition, was presented by Hernandez et al. [41], used for identification of celestial bodies in the sky. For a more general shape analysis, B-spline approximation can be used. Application of B-splines involves miscellaneous areas. An example thereof is research presented by Pedrosa et al. [42]. They propose a framework for three-dimensional (3D)-ultrasound segmentation in cardiology. B-spline shape representation is sometimes used in shape design optimization task [43].

Shape signature represents a shape by one dimensional function (e.g., distance from centroid, tangent angle, curvature), derived from contour points [36]. Application of shape signature involves a very broad spectrum of domains like traffic sign recognition [44], recognition of facial expressions [45], detection of anaemia blood cells [46], classification and sorting of agricultural products [47].

Fourier descriptors constitute an important class of global invariant features based on algebraic properties of Fourier transform [48]. They are considered to be very promising descriptors as they have the advantages of computational efficiency and attractive invariance properties [49]. They may be applied to solve very challenging identification tasks, including analysis and classification of shapes encountered in nature: Blood cells [50] or leaves [51]. In the applications similar to the latter, even better classification accuracy may be obtained using wavelet descriptors [52].

An interesting contour recognition method is elastic matching (also known as deformable template). It is one of methods used in optical character recognition systems (OCR) [53]. Its basic idea is to optimally match the unknown symbol against all possible elastic stretching and compression of each prototype. Therefore, it is also one of methods used in handwritten character recognition [54]. An interesting application of this method was presented by Attalla and Siy [55] in their shape recognition algorithm where elastic matching was employed as an auxiliary method when the shapes were partially occluded.

Among region-based shape representation techniques, there are Zernike moments [56] and Legendre moments, both belonging to a family of orthogonal functions, which allow the generation of non-redundant descriptors by the projection of an image onto an orthogonal basis [57].

Convex hull of a region is the smallest convex region containing the region in question. Convex hulls are used for multiple purposes, e.g., image registration and retrieval, image classification, and shape detection [58,59].

**Figure 1:**
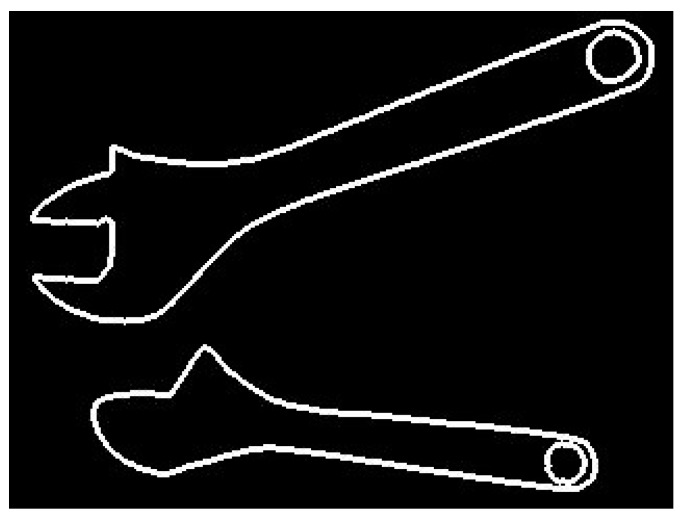
Shape differences between images of the same object.

Unfortunately, all those methods are not suitable for domain of industrial collaborative robotics (except some simple cases). This domain is very specific. On the one hand, contours of workpieces or tools handled by industrial robots usually consist of quite simple segments like lines, arcs, and sometimes Bezier curves, unambiguously determined by small sets of numerical parameters. Therefore, there is no need to apply methods used for recognition of ambiguous, non-rigid, or fuzzy shapes encountered in the nature (e.g., methods based on Fourier descriptors). On the other hand, the objects belonging to the same class may significantly vary in shape. Good examples are contours of adjustable wrenches, shown in Figure 1. 

The shape of adjustable wrench depends on how wide the wrench is open. Therefore, the use of methods, e.g., those based on signature matching, may lead to false classification (images of the same object would be classified to different groups). Seemingly, elastic matching may be a good solution. However, shape similarity between two workpieces or two tools does not necessarily indicate they belong to the same class. Also, the region-based methods, based on shape descriptors, may neglect small but critical differences in shape between objects belonging to different classes.

The problem of correct shape classification is a very challenging one in the case of industrial collaborative robotics as it is difficult to formulate the general rules which the humans employ (consciously or unconsciously), in order to rank the shapes into proper classes. Additionally, those rules may be strongly application-specific. Depending on the application, small differences in shape may be crucial, but sometimes the same or even much bigger differences may be irrelevant. Hence, we came to the conclusion that relying on human knowledge and experience is the best approach when formulating the rules for shape classification in individual applications. However, the formulation of those rules could be a very labor-intensive and time-consuming task. Therefore, there was a need to create appropriate tools to facilitate it. Users should be able to create and edit flexible contour templates, and thus, adjust image recognition systems to their needs. In this manner the customized task-oriented communication systems, between humans and collaborative robots, could be relatively easily developed. This conclusion was the basis for the flexible editable contour templates and FCD format, developed as a result of our research.

The most important advantage of our method consists in a new approach to template creation. The users are able to create flexible contour templates in an easy and transparent way. Those templates, based on the human experience and the specific character of an individual application, effectively combine two contradictory requirements that must be often fulfilled in order to properly recognize the objects in industrial environment: Broad (but properly targeted) flexibility for some parts of the template and strict precision requirements for the other ones.

It must be emphasized, that application of other methods is obstructed not so much by the fact they are not flexible enough but rather by the fact they cannot cope with the problem of these contradictory requirements. The philosophy standing behind them is simply different. Some types of objects simply cannot be successfully classified using those automatic methods that are not based on human experience (an example of such object is a traditional key where the key shank and the key bow may be of any shape but the key bit must be strictly determined in order to enable locking the door). In particular, it would be almost impossible to successfully apply those methods when there is a need to represent features of interest separately from features to be neglected (this issue will be described in detail in Section 3).

## 3. Flexible Editable Contour Templates

As objects (tools, workpieces etc.) belonging to one class may differ (sometimes even significantly) in shape and/or dimensions, it is necessary to provide flexibility of contour templates. However, it cannot be the art of flexibility used in elastic matching. Template flexibility must be strictly selective, according to the rules which are specific for individual applications. For example, some template dimensions may vary in a broad range but the other ones must be preserved. The rules may refer both to dimensions as to geometric relations between contour segments (e.g., perpendicularity, distance etc.). Another requirement is ability to create and modify the templates by the users in relatively effortless manner. Therefore, the rules must be expressed in a simple and transparent way. All those features are provided by FCD format used for template description.

**Figure 2:**
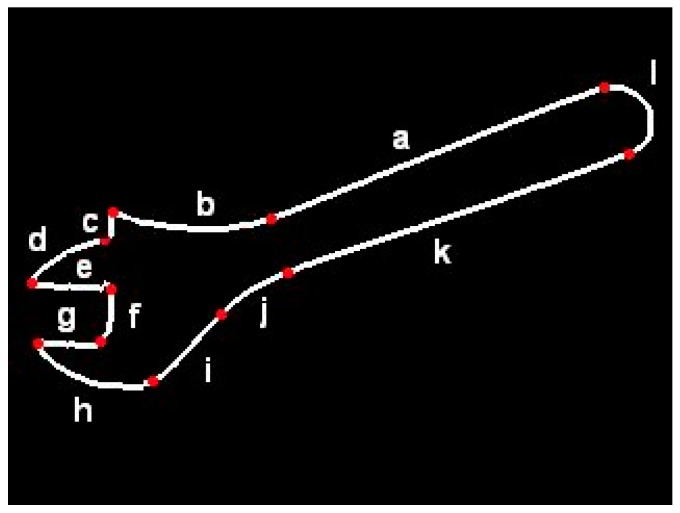
Visualization of flexible template “adjustable wrench”.

As industrial robots usually operate on objects whose contours consist mainly of relatively simple geometric entities, FCD format in its current version makes use of line segments, arcs, and restricted set of Bezier curves. There is obviously the possibility to extend this scope. A description of flexible template in FCD format has the following structure, presented below in extended Backus-Naur form:

flexible template= heading,contour description;
heading=‘#cnt’,[space],template name,new line;
template name=name;
contour description=statement | contour description,statement;
statement=direction description,new line | segment description,new line;
direction description=’go:’,direction,[‘/’,angle];
segment description=line description | curve description | virtual sement;
line description=’line:’,name,[‘/’,line specification];
curve description=curve type,’:’,name,‘/’,curve specification];
virtual segment=’virtual:’,name,[‘/’,line specification];
curve type=‘arc’ | ‘bezier’;
curve specification=arc specification | bezier specification;
line specification=length;
arc specification=curvature,[angle and radius];
bezier specification= curvature,[angle and radius],‘,’,weight;
angle and radius=[’,’,angle],[’,’,radius] | [’,’,radius],[’,’,angle];
curvature=’bend:’,direction;
length=’length:’,value;
angle=’angle:’,value;
radius=’radius:’,value;
weight=? value from the range (0,1> ?;
direction=’left’ | ‘right’;
value=variable name | [variable name,’=’],value specification;
value specification=single value | range of values;
range of values=lower limit,’,’,upper limit;
lower limit=single value;
upper limit=single value;
single value=?number? | ?arithmetic expression?
variable name=name;
name = letter | name,letter | name,digit | name,‘_’;
digit = ‘0’|‘1’|‘2’|‘3’|‘4’|‘5’|‘6’|‘7’|‘8’|‘9’;
letter = ? alphabetic character ?;
new line=?new line character?;
space=?space character?;

In order to provide an example of FCD-based contour description, the flexible template of adjustable wrench shown in Figure 2 is presented below.

# cnt adjustable wrench
line: a
arc: b/ bend: right, angle: alpha=1,45
go: left/ angle: beta=90,135
line: c
go: right/ angle: gamma=45,90
arc: d/ bend: left, angle: delta=1,90
go: left/ angle: epsilon=gamma-delta+90
line: e/ length: l
go: right/ angle: 90
line: f
go: right/ angle:90
line: g / length: l
go: left/ angle: epsilon
arc:h/bend:left,angle:zeta
line:i
arc:j/bend:right,angle:-alpha+beta+gamma-delta+zeta-180
line:k
arc: l /bend:left, angle:180

As can be seen, an individual FECT is a closed contour consisting of elementary segments defined in a flexible manner. Dimensions can be determined unambiguously or as the ranges of values. Contour description in FCD has a form “drawing instructions”, e.g., “Draw the straight line *a*, continue with the arc *b* which is bent to the left. The angle of this arc is up to 45 degrees. Then, turn left at angle of 90 to 135 degrees…” etc. It is similar to languages used in computer-aided machining (CAM) systems (e.g., APT language) for determining tool paths. However, in contrary to those languages, this description does not have a rigid character as it lets ranges and uses variables to be determined, as well as arithmetic expressions. Template description in FCD format is a sequence of statements determining direction of “drawing” (e.g., *go: right*), as well as shape and dimensions of individual segments (e.g. *line: e / length: l*). Geometric relations between contour segments are expressed indirectly by arithmetic expressions determining linear and angular dimensions. For example, perpendicularity of neighboring segments may be expressed by the use of statements like “*go: right / angle: 90*”. When relations between non-neighboring segments must be determined, those expressions may refer to variables introduced in previous statements. An example is expression determining the angle of arc “j” in the above template. It provides that lines “a” and “k” are parallel.

The use of ranges is another element of template flexibility. It lets classify objects of even significant shape differences to one group. On the other hand, this flexibility is strictly controlled.

Assigning names to the segments requires additional explanation. Generally, the segment names are not needed by contour classification algorithm. However, those names can be used later by the algorithm for calculation of robot gripper position when generating robot control instructions. They facilitate potential modification of flexible templates which are illustrated by images like that in Figure 2.

**Figure 3:**
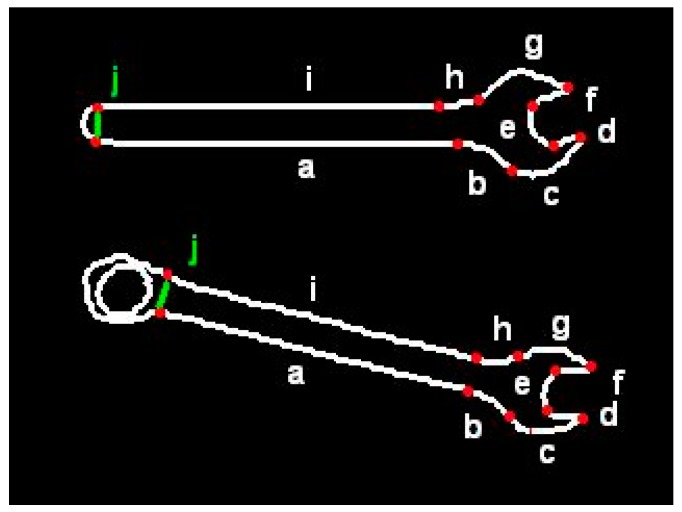
Visualization of flexible template containing a virtual segment.

In order to increase flexibility of the templates, virtual segments were introduced. They let classify the objects of quite different shapes to one group, under condition they possess certain key features. For example, the template presented below is valid for both wrenches shown in Figure 3:

#cnt wrench
line:a
arc:b/bend:right,angle:alpha=1,45
arc:c/bend:left,angle:beta,radius:r
go:left/angle:gamma=90,150
line:d/length:l
go:right/angle:90
line:e
go:right/angle:90
line:f/length:l
go:left/angle:delta=90,150
arc:g/bend:left,angle:epsilon
arc:h/bend:right,angle:-alpha+beta+gamma+delta+epsilon-360
line:i
go:left/angle:70,110
virtual:j

In a particular application it may be of no importance which of those two wrenches is recognized. From the human operator’s point of view both are appropriate because they are identical on one side. Whether it is open-end wrench or box-end wrench on the other side. The statement “virtual: j” in above template stands for any sequence of segments of contour, derived from image recognition system. Hence, when the user says: “Give me the wrench size twelve”, the robot will pass any of those wrenches, depending on which is currently available.

## 4. Shape Classification Algorithm

If the templates were rigid, classification of contours could be easily performed e.g. with the help of signature matching method. However, in our case the templates are flexible, even in a wide range. Therefore our contour identification algorithm consists of two consecutive steps:

- Try to roughly superimpose the flexible template on each contour derived from the image.
- When the first step is successful for one or more contours: compare the signatures of superimposed (adjusted) template and of contour(s) in question.

**Figure 4:**
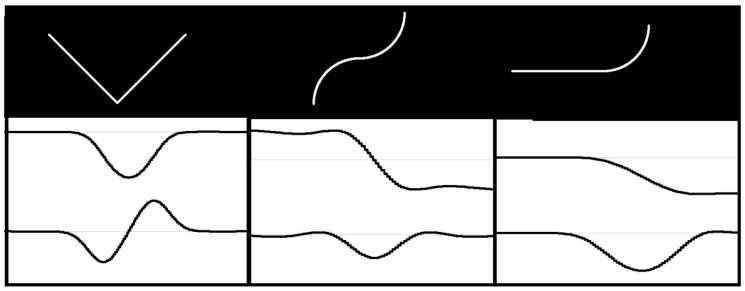
Typical feature points and corresponding signature plots (upper plot: curvature, lower plot: derivative).

In order to facilitate the first step, the template description in FCD format has the previously mentioned form of “drawing instructions”. However, those statements are usually ambiguous due to the indeterminate length of line segments, indeterminate radius or angle of arcs etc. As a result, the number of possible template instances is usually infinite. Superimposing (adjustment) of flexible template on a particular contour in the image may reduce the ambiguity level. However, the number of possible variants of adjustment is huge. In order to reduce it, contour feature points must be first extracted from the image. The contour feature points should correspond to template feature points, i.e., the points of the template, where the neighboring segments adjoin each another. It means the contours in the image must be searched for points where specific changes in curvature occur. Depending on the type of feature point, the first derivative of curvature function may be useful. Therefore, the shape signature in the form of curvature function, as well as its derivative, must be created for each contour in the image. The analysis of signature plots extract the feature points. The plots of curvature function, and its derivative for various types of feature points, are shown in Figure 4. The upper plot shows the curvature and the lower one – its derivative. We may distinguish between two types of feature points: Points of type 1 corresponding to local extrema of curvature function (left picture in Figure 4), and; whereas the points of type 2 correspond to local extrema of curvature derivative (middle and right picture in Figure 4).

As contours are derived from discrete binary images, the curvature function may change frequently and rapidly. This hinders the analysis. Therefore, contours must be first smoothed. It has some side effects, e.g., there will be no abrupt changes in curvature even at sharp corners (as it is the case in theoretical contours). It hinders exact recognition of corners and makes it impossible to distinguish them from little fillets. However, it has also a positive effect: Sharp corners are actually marked by three points (one of type 1 and two of type 2). This works like double-checking and provides the corner recognition to be performed correctly even when some feature points are not detected due to any reasons. On the other hand, two segments fluently passing into each other are marked with one point of type 2 only. If such a point is not detected for any reason, it may simply result in false classification of contour containing this point.

**Figure 5:**
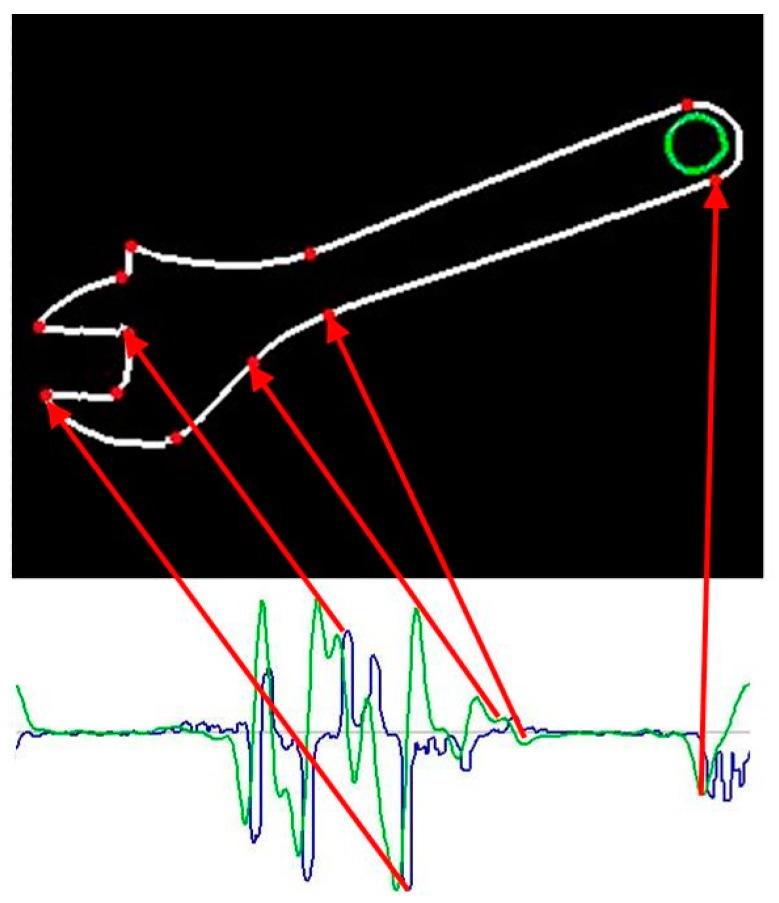
Sample feature points extraction based on signature analysis.

A sample contour of adjustable wrench and the plots of its signature are shown in Figure 5.

After contour feature points have been determined, the flexible template may be superimposed on the contour. Due to image distortions and inaccuracies, some recognized contour feature points may not correspond to any template feature points at all (redundant points). It means the number of detected feature points is greater than the number of segment endpoints in the template. It may result in a considerable number of possible variants of template adjustment. As full analysis of all possible variants is computation intensive, it is better to divide it into two phases: Rough and detailed matching. When negative results are obtained in the first phase, detailed matching does not have to be performed at all.

The matching algorithm uses an ordered set of vectors, describing the parameters of contour segments (exactly in the order the segments occur in contour). All possible variants must be taken into account, as a priori is not known, which contour segment corresponds to the first FECT segment. This could be computationally expensive, but due to division of the algorithm into phases, all variants must be taken into account only for rough matching. Some starting points may be eliminated at once, based on direction statements included in FECT. The detailed matching (which is computationally more expensive) must be performed generally for one variant only (in particular cases a few variants).

The rough matching refers to feature points only and neglects details of contour shape. In order to check if a given variant may result in successful superposition, only direction statements (e.g. “go: right”) and general curvature description (e.g. “bend: left”) are used. The general algorithm for rough matching is presented below:
**Algorithm 1:** Rough matchingInput: - Flexible template (T)      - * of contours in the image (C)* *Output: - Set of potentially matching contours (M)*      - *Set of starting points for each i^th^ matching contour (S_i_)*      - *Set of combinations of feature points for each m_i_ € M and s_ij_ € S_i_ (F_ij_)* *i ← 0% reset number of potentially matching contours* *for each c_k_ € C do*  *% create set P_k_ = (p_k_: p_k_ is feature point of k^th^ contour}*  *q ← get number of points belonging to c_k_*  *for n = 1..q do*   *crv_kn_ ← compute curvature for n^th^ point of c_k_*   *drv_kn_ ← compute derivative of curvature for n^th^ point of c_k_*  *end for*  *l ← 0% reset number of feature points for c_k_*  for n = 1..q do   *if crv_kn_ is local extremum then*    *l ← l + 1*    *type(p_kl_) ← 1% create feature point of type 1*    *x(p_kl_),y(p_kl_) ← get coordinates of n^th^ point of c_k_*   *end if*   *if drv_kn_ is local extremum then*    *l ← l + 1*    *type(p_kl_) ← 1% create feature point of type 2*    *x(p_kl_),y(p_kl_) ← get coordinates of n^th^ point of c_k_*   *end if*  *end for*  *% Attempts to superimpose template T on contour c_k_*  *i ← i + 1% increase number of potentially matching contours*  *m_i_ ← c_k_ % assume c_k_ as i^th^ matching contour m_i_ € M*
  *j ← 0% reset number of starting points for m_i_*  *for each p_kl_ € P_k_ do*    *j ← j + 1% increase starting point number*
    *s_ij_ ← p_kl_ % assume p_kl_ as j^th^ starting point s_ij_ € S_i_*
    *FPK ← create feature point combinations for j^th^ starting point*    *e ← 0% reset number of feature point combinations*    *for u = 1..|FPK| do*     *e ← e + 1% increase number of matching combinations*     *f_ije_ ← fpk_u_ % assume fpk_u_ € FPK as e^th^ combination f_ije_ € F_ij_*     *match ← true % auxiliary logical variable*     *for each template statement t_y_ € T do*      *if conditions imposed by t_y_ are not fulfilled by f_ije_ then*       *match ← false*      *end if*     *end for*     *if not match then*      *e ← e − 1*      *delete e ^th^ feature point combination f _kne_*     *end if*    *end for*    *if e = 0 then*     *j ← j − 1% decrease starting point number*    *end if*  *end for*  *if j = 0 then*    *i ← i − 1% decrease matching contour number*  *end if*
 *end for*

The second phase (detailed matching) is performed only for the variants for which positive result was obtained in the first phase. Its aim is to evaluate similarity between contour shape and the template using signature matching method. Signature differences are interpreted as the distance between points belonging to contour and corresponding points of the template. This distance is measured along the straight line perpendicular to the template. First, each template segment must be adjusted in order to coincide as close as possible with corresponding contour segment. This can be obtained by adapting such parameters, e.g., radii of arcs (when they are not fixed) in order to minimize the above mentioned distance. After all segments have been processed in this manner, the standard deviation of this distance for the whole contour is calculated as a measure of shape similarity. If this measure does not exceed a pre-determined value, the contour is considered to be identified. The general algorithm for detailed matching is presented below:
**Algorithm 2 Detailed matching** *Input: - Flexible template (T)*      - *Set of potentially matching contours (M)*      - *Set of starting points for each i ^th^ contour (S _i_)*      - *Set of feature point combinations for m_i_€M and s_ij_€S_i_ (F_ij_)*      - *Maximum acceptable error (err)* *Output: - Identified contour (I)*      - *Starting point for this contour (P)*      - *Set of feature points for identified contour (FP)* *v ← 0% reset variant number* *for each m_i_€M do*  *for each s_ij_€S_i_ do*   *for each f_ijk_€F_ij_ do*    *for each point pf_ijkl_€f_ijk_ do*     *adjust parameters of l^th^ segment of template T*    *end for*    *r ← 0% reset contour point number*    *for each pf_ijkl_€f_ijk_ do*     *n ← get number of points corresponding to l^th^ template segment*     *for q = 1..n do*      *r ← r + 1% increase contour point number*      *d_ijkr_ ← calculate distance between q^th^ point and the template*     *end for*    *end for*    *match ← false % auxiliary logical variable*    *mse_ijk_ ← calculate the mean squared error for d_ijkt_ where t = 1..r*    *if mse_ijk_ < err then*     *v ← v + 1*     *if v = 1 then*      *match ← true*     *else*
      *if mse_ijk_ < min_err then*       *match ← true*      *end if*     *end if*     *if match then*      *min_err = mse_ijk_ % min_err is the smallest error until now*      *I ← m_i_*      *P ← s_ij_*      *FP ← F_ijk_*     *end if*    *end if*   *end for*  *end for* *end for*

## 5. Experimental Results

**Figure 6:**
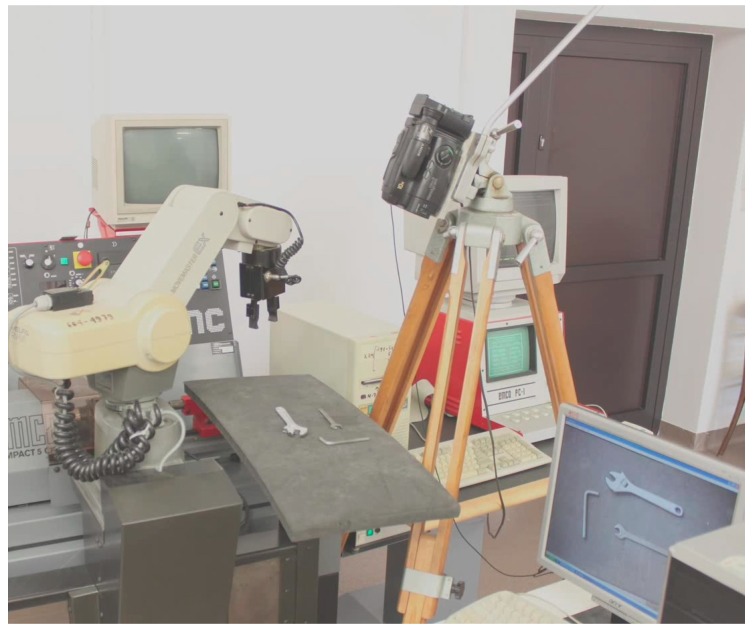
Robotized machining cell equipped with speech and image recognition systems.

In order to experimentally verify the results of our research, we developed and implemented a speech interface for communication with a robot being a part of flexible machining cell depicted in Figure 6.

This interface employs Microsoft SAPI speech engine. The sub-language of voice commands is defined using VCD (voice command description) format [15]. Sub-language description is available as supplementary data research data S1. The speech interface is integrated with an image recognition system which employs the method for contour identification described in the current paper. Machine tending is the robot’s basic role, but it may also cooperate with human operator during machine setup. In our experiment the task of the robot was to pass the tools required by the operator. As the operator has his hands engaged, he communicates with the robot by speech. The tools (e.g., various types of wrenches) are placed in random positions and they are in the field of view of a camera connected to image recognition system. Depending on the command uttered by the human operator, appropriate flexible contour template is matched against contours obtained from the image. The functioning of the whole system is presented in detail in Video S1 (Video1.mov file available as supplementary data). Flexible templates, used in this application, are available in Supplementary research data S2.

The effectiveness of our method can be measured using two parameters: Percentage of correctly classified contours (we will call it "recognition rate") and the time needed to recognize the contour. The former one is crucial for evaluation of the method, although it must be emphasized that the recognition rate does not necessarily reflect the quality of the object classification method, as it depends, not only on the method used, but also on several factors not related to the method itself, e.g., the better or worse workspace illumination, the material of objects to be recognized (e.g., images of metallic objects are often influenced by reflections), character of the background, image resolution, and many others.

As described previously, the most important advantage of our method consists in a new approach to template creation. The FECTs effectively combine two contradictory requirements that must be often fulfilled, in order to properly recognize the objects in industrial environment: Broad (but properly targeted) flexibility for some parts of the template and strict precision requirements for the other ones. This advantage has rather a qualitative character and it is not measurable. The issue cannot be reduced to the comparison between different methods based on their recognition rates.

The latter parameter (time needed to recognize the contour) is almost equally important as the former one because our method is expected to be used mainly in combination with speech-based communication between robot and human operator. Significant delays in the robot’s reaction to speech commands would make the whole system not very useful. 

It must be emphasized that high recognition rate and short reaction delays are two contradictory expectations. It results from potential existence of redundant points. As can be concluded from previous section, correct extraction of contour feature points is the key factor for reliable functioning of contour classification. As the feature points are the endpoints of contour segments, they are characterized by specific changes in contour curvature. The key problem is to properly adjust sensitivity of detection of those changes (we will call it “sensitivity level”). High sensitivity level provides high contour recognition rate but it results in detection of many redundant points due to inaccuracy and distortions of the image. When the number of feature points is increased, the number of passes of rough matching algorithm grows combinatorially. This may lead to substantial delays in robot reaction. 

In contrary, the low sensitivity level generally results in reduction of redundant points but it may cause some feature points not to be detected. This is equivalent to the failure of contour recognition.

In order to perform essential tests regarding recognition rate and recognition time, first the influence of image resolution on the number of redundant points was evaluated. An experiment was conducted with an object consisting of 17 segments of various types (the images used in the experiment as well as the corresponding signature plots are part of Supplementary research data S3). Recognition of this object was performed several times using the same template, but various image resolutions. Table 1 presents the results.

As can be seen, positive results have not been obtained for very low resolutions only. On the other hand, the test results show that a high resolution is neither necessary nor even desirable. As far as there are no unrecognized feature points, the algorithm functions fully reliably, independent of the resolution. However, the use of high resolution reveals and emphasizes small irregularities in the shape hence it generally results in an increased number of redundant points. This makes computing time longer and leads to delayed robot reactions. 

After appropriate image resolution had been determined for later experiments, the essential tests could be performed. We used six various templates of tools to be manipulated by robot in those tests. Images of those tools are available in Supplementary research data S4. Eight experiments were conducted using eight different sensitivity levels. Each experiment was conducted 10 times for various object positions and orientations i.e. each template was matched against 10 images of corresponding tool. The experiments are summarized in the results in Table 2: Recognition rate (in %) and the longest recognition time for a given template (in seconds). As far, as recognition time is concerned, it depends, not only on the method, but also on the equipment used for computation. In our case it was Windows PC computer with processor INTEL(R) CORE(TM) i3-7020 CPU @ 2.30 GHz. Application of more powerful processor would shorten the recognition time.

As can be seen, the test results are satisfactory. However, they also confirmed theoretical conclusions that high recognition rates and short reaction delays are two contradictory expectations. In order to provide 100% recognition rates and simultaneously avoid significant delays, appropriate measures must be undertaken, resulting in decrease in the number of redundant points. Fortunately, FECTs are created by humans who can adjust the templates to individual needs. The following simple practical guideline on flexible templates can be formulated: It is neither necessary nor desirable to include all shape details in the FECT, unless those details are crucial for distinction between objects. As may be concluded from discussion in Section 4, this refers in particular to those parts of contours which contain the feature points of type 2.

## 6. Discussion

The development of a novel method for contour identification based on flexible editable contour templates (FECT) is the main contribution of the research, presented in this paper, to the area of human-machine communication. This method helps to solve the challenging problem of shape classification that results from difficulties in automatic formulation of the rules that humans employ to rank the shapes into classes, corresponding to abstract terms used in task-oriented commands for the robots. Depending on the application, small differences in shape may be crucial, but sometimes, the same or even much bigger differences may be irrelevant. As explained in Section 2, existing methods could not be applied to cope with this problem. Our approach assumes that construction of the rules for shape classification should rely on user’s knowledge and experience. The rules must be constructed individually for each application. As it may be a labor-intensive and time-consuming task, we developed FCD (flexible contour description) format that helps to create and modify FECTs in an easy and transparent way. The users will be able to develop the customized vision-based task-oriented communication systems between humans and robots in relatively easy way.

Theoretical considerations were practically verified, as described in Section 5. A speech interface for the collaborative robot being a part of flexible machining cell has been developed and tested.

Although, research and tests, presented in this paper, proved our method to be useful, but further improvements should be introduced. The future plans comprise extension of FCD format in order to introduce description of optional subcontours. When using the current version, it is sometimes necessary to create several templates for one object when the shape of this objects depends on its state. For example, Supplementary research data S2 contains three templates for adjustable wrench (closed, open, wide open) because there are, not only quantitative, but also qualitative differences in its shape. This problem was partially solved by implementation of "virtual segments" (described in Section 3). They allow one template for even quite different contours sharing only few features. This refers also to various forms of one tool. However, virtual segments are neglected by matching algorithm, hence they cannot be used in all situations. The introduction of optional subcontours will ensure only one template is created for such objects (i.e., objects consisting of several moveable parts) also in situations when all elements of contours must be taken into account, in order to match the template properly.

Another improvement will consist in introduction of subroutines into FCD format. Subroutines will be used to describe the subcontours parametrically, hence, a number of different templates will be able to refer to them. It will be possible to create libraries of typical subcontours. In this way development of flexible templates will be facilitated.

As stated in the introduction, our final aim is a general solution for integration of industrially-oriented image and speech recognition systems. In the very next step, we will develop a method for construction of rules for identification of objects described by abstract terms and consisting of several contours. The object identification rules will be based on topological and geometrical relations between those contours. Subsequently, the previously developed VCD (Voice Command Description) format [15] will be extended, in order to include identification rules in voice command sublanguage description.

## Figures and Tables

**Table 1:** Influence of image resolution on the number of redundant points.

Image Resolution (kpix)	Redundant Points	Undetected Points
307	8	0
277	6	0
249	8	0
222	5	0
196	4	0
173	5	0
151	4	0
130	4	0
111	5	2
93	2	2
77	1	2

**Table 2:** Test results.

Sensitivity Level	Template 1	Template 2	Template 3	Template 4	Template 5	Template 6
**1**	70%	60%	70%	70%	100%	100%
	0.1s	0.05s	0.05s	0.05s	0.01s	0.01s
**2**	80%	60%	80%	80%	100%	90%
	0.1s	0.05	0.1s	0.03s	0.01s	0.01
**3**	90%	70%	90%	80%	100%	100%
	3.2s	0.01s	0.1s	0.4s	0.01	0.01s
**4**	100%	90%	100%	100%	100%	100%
	2.0s	0.7s	0.15s	1.7s	0.1s	0.1s
**5**	100%	80%	90%	90%	100%	100%
	5.2s	0.2s	0.15s	0.4s	0.05s	0.05s
**6**	100%	100%	100%	100%	100%	100%
	8.0s	1.9s	0.4s	3.2s	0.15s	0.15s
**7**	100%	100%	100%	100%	100%	100%
	10.0s	3.0s	0.8s	5.1s	0.25s	0.25s
**8**	100%	100%	100%	100%	100%	100%
	15.0s	4.5s	1.2s	7.5s	0.4s	0.4s

## Supplementary Materials

The following are available online at https://www.mdpi.com/1424-8220/20/6/1773/s1, Video S1: Video1.mov, Research data S1: research data 1.doc, Research data S2: research data 2.doc, Research data S3: research data 3.doc, Research data S4: research data 4.doc.
