# Supplementary material for: Object Identification for Task-Oriented Communication with Industrial Robots
Source: Sensors (Basel). 2020 Mar 23;20(6):1773. doi: 10.3390/s20061773 (PMC7147712; doi:10.3390/s20061773)
Supplement: Supplementary file 1 [file sensors-20-01773-s001.zip › research data 1.doc]

**Sublanguage of voice commands in VCD format (file RCIM.VCD):**

#com open

?robot open ?the gripper

#com close

?robot close ?the gripper

#com put down

?robot put *wrench down

#def wrench

?the wrench

it

#com give me

?robot *giveme *tool :p2

#def giveme

give me

reach me

#def tool

?the *type wrench :p1

#def type

adjustable :1

open end :2

allen :3

**Results of automatic translation of sublanguage description into XML format supported by Microsoft SAPI (file RCIM.XML):**

<grammar langid="409">

<rule name="RCIMOPEN" toplevel="active"><l>

<p>

<o disp="[ROBOT]">ROBOT</o>

OPEN

<o disp="[THE]">THE</o>

GRIPPER

</p>

</l></rule>

<rule name="RCIMCLOSE" toplevel="active"><l>

<p>

<o disp="[ROBOT]">ROBOT</o>

CLOSE

<o disp="[THE]">THE</o>

GRIPPER

</p>

</l></rule>

<rule name="RCIMPUT DOWN" toplevel="active"><l>

<p>

<o disp="[ROBOT]">ROBOT</o>

PUT

<ruleref name="RCIMWRENCH"/>

DOWN

</p>

</l></rule>

<rule name="RCIMWRENCH"><l>

<p>

<o disp="[THE]">THE</o>

WRENCH

</p>

<p>

IT

</p>

</l></rule>

<rule name="RCIMGIVE ME" toplevel="active"><l>

<p>

<o disp="[ROBOT]">ROBOT</o>

<ruleref name="RCIMGIVEME"/>

<ruleref name="RCIMTOOL"/>

</p>

</l></rule>

<rule name="RCIMGIVEME"><l>

<p>

GIVE

ME

</p>

<p>

REACH

ME

</p>

</l></rule>

<rule name="RCIMTOOL"><l>

<p>

<o disp="[THE]">THE</o>

<ruleref name="RCIMTYPE"/>

WRENCH

</p>

</l></rule>

<rule name="RCIMTYPE"><l>

<p>

ADJUSTABLE

</p>

<p>

OPEN

END

</p>

<p>

ALLEN

</p>

</l></rule>

</grammar>
