# Supplementary material for: Object Identification for Task-Oriented Communication with Industrial Robots
Source: Sensors (Basel). 2020 Mar 23;20(6):1773. doi: 10.3390/s20061773 (PMC7147712; doi:10.3390/s20061773)
Supplement: Supplementary file 1 [file sensors-20-01773-s001.zip › research data 2.doc]

**Flexible templates of tool contours in FCD format:**

Open end wrench

**a**

**b**

**d**

**e**

**f**

**g**

**h**

**i**

**j**

**c**

#cnt open_end

line: a

arc: b/ bend: right, angle: alpha=1,45

arc: c/ bend: left, angle: beta, radius: r

go: left / angle: gamma=90,150

line: d/ length: l

go: right/ angle: 90

line: e

go: right/ angle: 90

line: f/ length: l

go: left/ angle: delta=90,150, radius: r

arc: g/ bend: left, angle: epsilon

arc: h/ bend: right, angle: –alpha+beta+gamma+delta+epsilon-360

line: i

arc: j/ bend: left, angle:180

Allen wrench

**a**

**b**

**d**

**e**

**f**

**g**

**h**

**c**

#cnt allen

line: a / length: la

arc: b / bend: right, angle: 90

line: c / length: lc

go: left / angle: 90

line: d

go: left / angle: 90

line: e / length: lc

arc: f / bend: left, angle: 90

line: g / length: la

go: left / angle:90

line: h

Adjustable wrench (open)

**a**

**b**

**c**

**d**

**e**

**f**

**g**

**h**

**i**

**j**

**k**

**l**

#cnt adjustable_open

line: a

arc: b/ bend: right, angle: alpha=1,45

go: left/ angle: beta=90,135

line: c

go: right/ angle: gamma=45,100

arc: d/ bend: left, angle: delta=30,90

go: left/ angle: epsilon=gamma-delta+90

line: e/ length: l

go: right/ angle: 90

line: f

go: right/ angle:90

line: g / length: l

go: left/ angle: epsilon

arc: h/ bend:left, angle:zeta

line: i

arc: j/ bend:right, angle:-alpha+beta+gamma-delta+zeta-180

line: k

arc: l /bend:left, angle:180

Adjustable wrench (closed)

**a**

**b**

**c**

**d**

**h**

**i**

**j**

**k**

**l**

#cnt adjustable_closed

line: a

arc: b/ bend: right, angle: alpha=1,45

go: left/ angle: beta=90,135

line: c

go: right/ angle: gamma=45,100

arc: d/ bend: left, angle: delta=30,90

go: left/ angle: 2*gamma-2*delta

arc: h/ bend:left, angle:zeta

line: i

arc: j/ bend:right, angle:-alpha+beta+gamma-delta+zeta-180

line: k

arc: l / bend:left, angle:180

Adjusable wrench (wide open)

**a**

**b**

**d**

**e**

**f**

**g**

**h**

**i**

**j**

**k**

**l**

#cnt adjustable_wide

line: a

arc: b/ bend: right, angle: alpha=1,45

go: left/ angle: beta=90,135

go: right/ angle: gamma=45,100

arc: d/ bend: left, angle: delta=30,90

go: left/ angle: epsilon=gamma-delta+90

line: e/ length: l

go: right/ angle: 90

line: f

go: right/ angle:90

line: g / length: l

go: left/ angle: epsilon

arc: h/bend:left, angle:zeta

line: i

arc: j/ bend:right, angle:-alpha+beta+gamma-delta+zeta-180

line: k

arc: l/ bend:left, angle:180
