# Supplementary material for: Object Identification for Task-Oriented Communication with Industrial Robots
Source: Sensors (Basel). 2020 Mar 23;20(6):1773. doi: 10.3390/s20061773 (PMC7147712; doi:10.3390/s20061773)
Supplement: Supplementary file 1 [file sensors-20-01773-s001.zip › research data 3.doc]

**Sample images and corresponding signature plots:**

Curvature – blue plot

Derivative – green plot

Red arrow – starting point

Adjustable wrench

Allen wrench

Open end wrench

Test shape (various image resolutions according to Table 1 in the paper)

Resolution 307 kpix

Resolution 277 kpix

Resolution 249 kpix

Resolution 222 kpix

Resolution 196 kpix

Resolution 173 kpix

Resolution 151 kpix

Resolution 130 kpix

Resolution 111 kpix

Resolution 93 kpix

Resolution 77 kpix
