# Supplementary material for: Object Identification for Task-Oriented Communication with Industrial Robots
Source: Sensors (Basel). 2020 Mar 23;20(6):1773. doi: 10.3390/s20061773 (PMC7147712; doi:10.3390/s20061773)
Supplement: Supplementary file 1 [file sensors-20-01773-s001.zip › research data 4.doc]

**Images of tools used in experiments**

**Tool 1:**

Binarized images:

**Tool 2:**

Binarized images:

**Tool 3:**

Binarized images:

**Tool 4:**

Binarized images:

**Tool 5:**

Binarized images:

**Tool 6:**

Binarized images:
